# Community health workers’ quality of comprehensive care: a cross-sectional observational study across three districts in South Africa

**DOI:** 10.3389/fpubh.2023.1180663

**Published:** 2023-12-14

**Authors:** Olukemi Babalola, Jonathan Levin, Jane Goudge, Frances Griffiths

**Affiliations:** ^1^Center for Health Policy, University of the Witwatersrand Faculty of Health Sciences, Johannesburg, South Africa; ^2^Division of Epidemiology and Biostatistics, University of the Witwatersrand Faculty of Health Sciences, Johannesburg, South Africa; ^3^University of Warwick Medical School, Coventry, United Kingdom

**Keywords:** community healthcare worker, quality-of-care, assessment tool, comprehensive care, performance

## Abstract

**Background:**

Community healthcare worker (CHW) training programs are becoming increasingly comprehensive (an expanded range of diseases). However, the CHWs that the program relies on have limited training. Since CHWs’ activities occur largely during household visits, which often go unsupervised and unassessed, long-term, ongoing assessment is needed to identify gaps in CHW competency, and improve any such gaps. We observed CHWs during household visits and gave scores according to the proportion of health messages/activities provided for the health conditions encountered in households. We aimed to determine (1) messages/activities scores derived from the proportion of health messages given in the households by CHWs who provide comprehensive care in South Africa, and (2) the associated factors.

**Methods:**

In three districts (from two provinces), we trained five fieldworkers to score the messages provided by, and activities of, 34 CHWs that we randomly selected during 376 household visits in 2018 and 2020 using a cross-sectional study designs. Multilevel models were fitted to identify factors associated with the messages/activities scores, adjusted for the clustering of observations within CHWs. The models were adjusted for fieldworkers and study facilities (*n* = 5, respectively) as fixed effects. CHW-related (age, education level, and phase of CHW training attended/passed) and household-related factors (household size [number of persons per household], number of conditions per household, and number of persons with a condition [hypertension, diabetes, HIV, tuberculosis TB, and cough]) were investigated.

**Results:**

In the final model, messages/activities scores increased with each extra 5-min increase in visit duration. Messages/activities scores were lower for households with either children/babies, hypertension, diabetes, a large household size, numerous household conditions, and members with either TB or cough. Increasing household size and number of conditions, also lower the score. The messages/activities scores were not associated with any CHW characteristics, including education and training.

**Conclusion:**

This study identifies important factors related to the messages provided by and the activities of CHWs across CHW teams. Increasing efforts are needed to ensure that CHWs who provide comprehensive care are supported given the wider range of conditions for which they provide messages/activities, especially in households with hypertension, diabetes, TB/cough, and children or babies.

## 1 Introduction

Community healthcare workers (CHWs), with limited training, are the focus in many low and middle-income countries’ (LMICs) attempts to accomplish the goal of universal health coverage of providing accessible, acceptable, and affordable healthcare services to vulnerable groups (1–3). CHW programs have historically focused on single diseases and conditions (mostly around maternal and child health, MCH) (2, 4). However, increasingly, they are now focusing on a more expanded range of diseases and people (including subpopulations with communicable and non-communicable diseases), towards more comprehensive care (2, 4–10). CHW programs in LMICs focusing on comprehensive care include Ethiopia’s Health Extension Worker Program (HEP), India’s Accredited Social Health Activist (ASHA), Pakistan’s Lady Health Worker Program (PLHW), Malawi’s Health Surveillance Assistants (HSA), government-employed community health extension workers (CHEWs) in Kenya, CHWs in Rwanda, and the community health assistants (CHAs) in Zambia (11–17). Although there is no specific definition for what constitutes ‘comprehensive care’, programs where a single CHW provides a “full range of services,” including preventive, promotive, and/or some curative care at the most peripheral level of the primary healthcare (PHC) system, may be considered ‘comprehensive,’ as discussed in a recent review (5). For example, Ethiopia’s 16 HEP healthcare packages cover family health, disease prevention/control and hygiene, and environmental sanitation (18). The ASHA comprehensive care package includes diagnosis of health conditions (e.g., malaria, tuberculosis, diarrhea); provision of drugs and referrals; reproductive, maternal, and newborn care; and nutritional counseling and health education (19). The PLHW program provides care to rural and urban slum communities on MCH (maternal antenatal care, birth record keeping, child development, family planning), promotive and preventive educational services (checking blood pressure, body temperature), treatment of minor diseases (childhood diarrhea, respiratory infections), and health education/promotion/referrals (20, 21). However, since these comprehensive CHW programs rely on a healthcare worker cadre with limited training, long-term or ongoing assessment is necessary to identify gaps in CHW competency within their routine work environment (1).

South Africa has had a unifying national CHW program framework since 2011, which is prioritized in the South African National Department of Health (NDoH) re-engineering of PHC (22). Alongside other healthcare providers, CHWs constitute the national Ward-based Primary Healthcare Outreach Team (WBPHCOT), in a largely comprehensive program, with CHWs serving a ‘generalist’ role (23). The activities of WBPHCOT include “health promotion, primary prevention of disease, healthy behavior counselling, treatment adherence counseling, and secondary disease prevention through basic screening” (24). The team is required to provide “appropriate referral and basic therapeutic, rehabilitative, and palliative care services.” The CHWs undergo two 10-day phase 1 curriculum-based training courses followed by a practicum, and a 5-day phase 2 skills-based training course, with a practical skill component (24, 25). However, implementation of the WBPHCOT program is uneven across districts and provinces, with variability in CHW service delivery (26, 27). Differences across provinces and districts in CHW activities occur regarding whether or not the CHWs ‘checked in’ at healthcare facilities before and after household visits, their hours of work, the required number of households to visit daily, whether or not routine weekly supportive training is carried out, and whether oversight supervision functions are carried out (27). These differences underscore the need to assess CHW competency, across districts, in South Africa.

There is increasing literature on essential and standardized metrics and validated indicators for assessing CHW competency; however, evidence suggests difficulty with assessing them within comprehensive care (28–30). Assessing CHW performance based on all subcomponents of a comprehensive care package is challenging (30, 31). This is because both structural (linkage with health system/community, assessment of available supplies and resources) and process measures (communication competency, compliance with standards of care and procedures, and activities related to care) are needed, as proposed by Donabedian (32–34).

CHW competency is assessed using several methods and tools. These include direct observation checklists either by supervisors, trained experts, or field investigators; a self-administered questionnaire; and administrative data (35–38). Their applications have mostly focused on subcomponents of comprehensive care packages. For example, Madagascar’s community health volunteers (CHVs) comprehensive care package included child and maternal health, case management of children <5 years, and reproductive health counseling and family planning (RH/FP) (35). However, using expert observers and gold standard evaluators, the CHVs were assessed on either case management of children <5 years or RH/FP. In South Africa, an assessment of CHWs’ knowledge and confidence using a self-administered questionnaire showed adequate confidence of CHWs in several activities (giving prescribed medications and managing clients with diabetes) and low knowledge and confidence levels in managing clients with hypertension (38). However, the findings were limited because of the lack of a validated tool. Another South African study that used a validated checklist based on audio recordings revealed, on average, good communication practices (active listening and active delivery) during home visits (39). However, only communication scores were reported, leaving out other skills of CHWs.

*S*tudies assessing factors influencing the performance of comprehensive care provided by CHWs within the household settings are also scarce. Kawakatsu et al. reported that performance was associated with CHWs’ individual-level (marital status and educational level), supervisor-level (number of supervisions received), and community-level factors (household size) (40). Sociodemographic factors (including age, sex, and education) also influence CHW performance (5, 41). Recently, in Kenya, Njororai et al. investigated the demographic and environmental factors affecting CHW performance (42). Using a self-reported questionnaire-based survey among 309 mixed cadre CHWs (volunteers, CHEWs who supervise CHWs, and retired CHWs), they found that having a secondary education and poor communication skills influenced performance. In these previous studies, while Kawakatsu et al. assessed performance using reports submitted in the previous 6 months by CHWs to their supervisors, Njororai et al.’s performance (categorized as poor/better) assessment was based on contextual information including whether the CHWs were available, participated, or were responsive to their work (40, 42).

Therefore, there is a need for research that assesses the factors influencing CHW performance related to their work during household visits using a validated tool. We developed an observational checklist, the quality-of-care assessment tool for CHW summative assessment in South Africa, in a previous study (43, 44). As previously reported, we developed the tool to account for the work of South African CHWs on a daily basis (before they leave the healthcare facility, upon entry into a household, and during the household visit) (Table 1) (28). A fieldworker utilizes our tool to record observed activities/messages and communication items to compute an aggregate score for each. Our evaluation of this tool suggests good utility, high validity (face and content) for the quality of messages and communication, and good interrater reliability for messages but not for communication scores (28). In this study, using this validated tool, we sought to answer the question “What factors are associated with the messages/activities scores of CHWs who provide comprehensive care in South African districts?.” Our study aimed to determine the performance (messages/activities) scores of CHWs who provide comprehensive care and determine what factors explain the scores. In this study, we focus on CHW household visits. This is important because the activities of CHWs during household visits often go unsupervised and unassessed.

**Table 1:** The structure of the quality-of-care assessment tool.

**Point when recordings/scores are indicated in the tool**	**Sections of the tool**
Before setting out	Items in the CHW bag including the list of equipment available for use during the household visit
Just before and on entry to a household (CHW seeks permission for the observer to observe the household visit)	The plan for the current household visit, which requires a ‘yes/no’ response.
	CHW communication skills, including attention to household members’ privacy and confidentiality, which requires a ‘yes/no’ response.
When performing activities or providing messages during household visit	Household members’ conditions and the messages/activities delivered by the CHW, which requires a ‘yes/no’ response.
	CHW communication skills, including attention to household members’ privacy and confidentiality, which requires a ‘yes/no’ response.
After leaving the household	Factors that could prevent a CHW from delivering good quality care
	CHW communication skills including attention to confidentiality

CHW, community health worker.

## 2 Methods

### 2.1 Study setting

The study was undertaken in two districts of Gauteng Province and one district of Mpumalanga Province, South Africa. Sedibeng District has approximately 1,039,908 people in 330,326 households, 12.3% live in informal households in shacks made of plastic and corrugated iron, while others live in brick houses, mainly provided by the government (45). Approximately half (50.7%) of the population are unemployed, live in poverty (48.5%), and depend on the government for social grants (45). Our two study facilities are located approximately 30 km from the district center. Tshwane District has approximately 3,555,741 people in 1,136,876 households; 22.6% are unemployed, 29.6% live in poverty, and 16.4% live in informal households (46). Our study facility is located approximately 32 km from the district center. Ehlanzeni District, in Mpumalanga Province, has approximately 1,856,753 people in 514,000 households; almost half (47.8%) do not receive an education at all, and 5.6% live in informal households (47). Approximately 43.4% of the population are unemployed, while 67.8% live in poverty (47). Our two study facilities are located approximately 138 and 126 km, respectively, from the district center.

### 2.2 Study design

This study forms part of a program of research reported elsewhere (43, 48). We undertook two cross-sectional surveys and combined the data for this study:

1. We collected data in 2017 in two facilities (A and B) in Sedibeng District.
2. We collected data in 2020 in two facilities (C and D) in Ehlanzeni District, Mpumalanga Province, and one facility (E) in Tshwane District, Gauteng Province.

The COVID-2019 pandemic limited the CHW observation to one facility in Tshwane District. We complied with the necessary COVID-19 prevention protocols during the study.

### 2.3 The CHW teams and their outreach team leaders

The outreach team leaders (OTLs) for the CHW teams included two enrolled nurses (ENs, having completed a 2-year nursing course), one per facility in Sedibeng District; four professional nurses (PNs, trained in PHC and community nursing), two per facility in Ehlanzeni District; and two PNs + one EN (Tshwane District). Their average nursing years of experience ranged from 2.4 to 4.5 years. Most of the OTLs were from the local communities and had undergone the WHBPCOT training. However, the PN OTLs are also tasked with providing PHC services in the clinic, limiting opportunities for supportive supervision. In one of the districts, one OTL who was an EN was not local. This OTL lacked confidence in providing supervisory support (48). Furthermore, facility E, including the WHBPCOTs, was designated a National Health Insurance pilot site in 2012 and underwent health system strengthening interventions focused on the PHC level (49).

### 2.4 Sampling methods

This study included all CHWs in the WHBPCOT program who worked in the three districts. Inclusion criteria were all the CHWs in each facility who were available for two full weeks of household visits to provide comprehensive care and were willing to participate. In any of the facilities, any CHWs who were not available for part or all of the 2 weeks of household visits and any CHWs who were providing home-based care only (i.e., providing bed baths, wound dressing, and cleaning support largely for the sick and non-sick older adults) were not considered eligible for the study. Random sampling was used to select each CHW for observation. We negotiated with the OTLs to ensure that the randomly selected CHWs focused on home visits. Three CHWs were randomly selected for each 2-weekly observation per cycle of observations for a total of four cycles (facilities A and B) and two cycles (facilities C, D, and E). Households were not selected, rather, the normal schedules of CHW pairs under the guidance of their OTLs were used to determine the household where the observation took place.

### 2.5 Quality-of-care tool

During household visits, to guide the non-clinically experienced but trained fieldworkers to complete our quality-of-care tool, we developed a fieldwork manual (44). The fieldworkers assign scores of ‘0’ or ‘1’ for messages/activities performed while observing CHWs during household visits using the tool. During household visits, the CHWs may perform messages/activities for adults, the older adult (sick and non-sick), pregnant women, and children and babies with a need. CHWs may also attend to children and babies in their absence by obtaining information regarding their immunization, nutrition, and HIV exposure status from caregivers/parents. Table 2 shows how the tool is used to derive performance (messages/activities) scores for a hypothetical ‘Household 1’ with hypertension and diabetes.

**Table 2:** Deriving the messages/activities score for a hypothetical ‘Household 1.’

**Household 1**									
**Conditions**	**Present (Yes = 1/ No = 0)**	**Messages/activities per condition**							**Aggregate score**
**Hypertension**		Lifestyle advice	Taking medication	Measuring BP	Access to medication				
	**1**	**1**	1	0	1				3/4 = 75.0
**Diabetes**		Lifestyle advice	Taking medication	Measuring BP	Access to medication		Glucose	Foot care	
	1	0	1	0	1		0	0	3/6 = 50.%
**Under 5**		Road To Health Card	Feeding/diet	Attending clinic	Looking at child				
								
**Under 6 months**		Mother’s health	Caring for the baby	HIV-related	Feeding	RTC	Attending the clinic	Looking at/examining baby	
								
**HIV**	1	Lifestyle advice	Taking medication	Access to medication/going to the clinic					
		1	1	1					3/3 = 100.0
**Persistent cough**		Screen for TB							
								
**TB**		Lifestyle advice	Taking medication	Access to medication /going to the clinic					
								
		Lifestyle advice	HIV status	Attending clinic/danger signs	Preparation of birth/baby				
**Pregnant women**									
		Advice							
**Other illness or potential illness**									
		Check up							
**Total number of conditions in household**	3								75% + 50% + 100% = 225/3
**Household message score = (observed/expected hypertension messages) ± (observed/expected diabetes messages) total number of conditions in household**									75.0%

BP, blood pressure; RTC, road-to-health card; TB, tuberculosis.

### 2.6 Data collection

The data collection approach in our previous study in Sedibeng District has been reported (43, 48). Briefly, in facilities A and B, in 2017, three trained fieldworkers utilized the paper-based form of our assessment tool to score the messages/activities and communication items of 12 and 10 randomly selected CHWs during 147 and 124 household visits, respectively. For facilities C, D, and E, in 2020, two trained fieldworkers utilized our tool to score the messages/activities of 2, 4, and 6 randomly selected CHWs during 40, 51, and 14 household visits, respectively.

For each randomly selected CHW (and partner), a brief interview was first used to obtain written consent, demographic details, content of CHW bags, and plans for the day. Within the households, the rest of the data collection was done through observation. The data collected while observing a total of 34 CHWs who provided services to 526 clients were captured separately by two fieldworkers in REDCap^1^ to minimize errors. Author OB then verified agreements between the two captured data. All the discrepancies were resolved by rechecking the data on the completed hard copy of the tool. The finalized data were then exported into Stata 17 (Stata Statistical Software: Release 17. College Station, TX: StataCorp LP) and merged for data cleaning and analysis. We had no details on three CHWs who were ineligible during random selection (because they had been on leave at the time of selection). However, after their return to work, they were paired with an eligible CHW (we had no control over the pairing). The messages/activities data of these three CHWs (in 36 households) were therefore excluded from the analysis.

### 2.7 Outcome

Our dependent variable, quality-of-care messages/activities score (hereafter messages/activities score), was derived as an aggregate score and defined as follows:

Messages/activities score: this is the number of observed messages given and actions undertaken divided by the number of expected messages. The messages/activities score was calculated for each condition (e.g., hypertension, diabetes, or HIV) and aggregated by household (Table 2).

### 2.8 Explanatory variables

- CHW*-*related characteristics: age, education level, and phases of CHW training attended and/or passed
- CHW activities: duration of visit (in minutes), normal frequency of visits, when CHW last visited the household, planned activities for this visit
- Household-related factors: household size (number of people per household); number of people with either hypertension, diabetes, HIV, TB, or cough; the presence of an infant/young child; and the presence of a pregnant person in the household
- Other contextual visit factors: whether there were ‘disruptions’ during the visit that could potentially affect CHW competency during the visit that were beyond the control of the CHW, e.g., baby crying, television noise, presence of visitors

### 2.9 Statistical analysis

Descriptive statistics are expressed as mean ± standard deviation (SD) for continuous variables and frequency and percentages for categorical variables. We performed one-way analysis of variance to compare messages/activities scores by district, CHW-related characteristics, CHW activities, household-related factors, and other contextual factors. Multilevel models were fitted to identify factors associated with messages/activities score, adjusting for the clustering of observations within CHWs. In the models, both fieldworker and facility were included as fixed effects due to the small number of each (there were only five fieldworkers and five facilities). CHW-related variables and contextual and household-related factors were investigated. In building the model, we used a backward elimination approach, whereby after we included all the variables in one model, we removed each variable from the model one by one, starting with the least significant, and then each model was refitted until all the variables that were significant at *p* < 0.1 were retained (50). To select the best model for predicting messages/activities scores, we evaluated the prediction errors using the principle of Akaike information criterion (AIC) minimization. In each model fitted, AIC imposes a penalty of 2p (where p is the number of predictors in the model) against the model deviance (− 2* log-likelihood), for each additional variable. We selected the model that minimized AIC as the best prediction model. In each model, we retained ‘facility’ as a variable, regardless of the level of significance. We reported the mean messages/activities scores and their 95% confidence intervals (95% CI). *p* < 0.05 was considered to be significant.

### 2.10 Ethical consideration

The Human Research Ethics Committee (Medical) of the University of the Witwatersrand, Johannesburg (approval number M190933), the respective ethics committee of participating Ehlanzeni (MP-2020001-002), and Tshwane Districts Research Committees (GP-202001-012) approved this study protocol. The participants provided their written informed consent to participate in this study and each CHW sought and obtained verbal consent from each head of household before the fieldworker was allowed to enter to perform observations.

## 3 Results

### 3.1 Characteristics of households and household members included in this study

The characteristics of 376 households and 526 household members who engaged with the CHWs are shown in Table 3. The mean number (standard deviation [SD]) of household members per visit was 1.8 (SD, 0.98), the CHWs mostly engaged with women (*n* = 369, 72.4%), individuals aged over 60 or who were retired or were older adults (*n* = 253, 48.1%), and those living in brick houses (*n* = 284, 75.5%).

**Table 3:** Description of household members who engaged with the CHW (for children, engagement may have been via caregivers/parent only).

**Characteristics**	**Total**		**Facility A**		**Facility B**		**Facility C**		**Facility D**		**Facility E**	
**Number of household members who engaged with CHWs (n, %)**	526	100.0	184	35.0	195	37.1	63	12.0	66	12.6	18	3.4
**Number of households visited (*n*, %)**	376	100.0	147	39.1	124	32.8	40	10.6	51	13.6	14	3.7
**Mean (standard deviation) and range of household size per CHW visit**	1.77 (0.98)	1–5	1.49 (0.74)	1–4	2.02 (1.05)	1–5	2.17 (1.31)	1–5	1.55 (0.79)	1–4	1.44 (0.51)	1–2
**Sex (*n*, %)**												
Male	145	27.6	51	27.7	63	32.3	13	20.6	14	21.2	4	22.2
Female	381	72.4	133	72.3	132	67.7	50	79.4	52	78.8	14	77.8
**Age category (*n*, %)**												
Baby under 6 months	21	4.0	4	2.2	9	4.6	7	11.1	0	0.0	1	5.6
Pre-school child	44	8.4	9	4.9	20	10.3	12	19.1	3	4.5	0	0.0
School-age child/adolescent	11	2.1	2	1.1	7	3.6	0	0.0	2	3.0	0	0.0
Adult <60	197	37.4	47	25.5	90	46.2	28	44.4	30	45.5	2	11.1
Over 60/retired/older adult	253	48.1	122	66.3	69	35.4	16	25.4	31	47.0	15	83.3
**Type of housing (*n* = 376)**												
Informal (shacks)	92	24.5	20	13.6	72	58.1	0	0.0	0	0.0	0	0.0
Formal (brick)	284	75.5	127	86.4	52	41.9	40	100.0	51	100.0	14	100.0

CHW, community health worker.

Descriptions of the conditions of household members who engaged with the CHWs (for children, engagement may have been via their caregivers/parents only) are shown in Table 4. In all the facilities, the CHWs mostly encountered hypertension (*n* = 218, 41.4%). Across districts, most CHWs encountered only one condition per household (*n* = 263, 50.0%).

**Table 4:** Description and burden of conditions of household members who engaged with the CHW (for children, engagement may have been via their caregivers/parent only).

**Characteristics**	**Total**		**Facility A**		**Facility B**		**Facility C**		**Facility D**		**Facility E**	
**Number of household members who engaged with CHWs (*n*, %)**	526	100.0	184	35.0	195	37.1	63	12.0	66	12.6	18	3.4
**Number of households visited (*n*, %)**	376	100.0	147	39.1	124	32.8	40	10.6	51	13.6	14	3.7
**Condition (*n*, %)** ^**a**^												
Hypertension	218	41.4	124	67.4	48	24.6	16	25.4	19	28.8	11	61.1
Diabetes	73	13.9	31	16.9	21	10.8	8	12.7	8	12.1	5	27.8
HIV	72	13.7	16	8.7	26	13.3	15	23.8	15	22.7	0	0.0
Tuberculosis	17	3.2	5	2.7	8	4.1	0	0.0	4	6.1	0	0.0
Other chronic	42	8.0	16	8.7	9	4.6	9	14.3	7	10.6	1	5.6
Persistent cough	29	5.5	22	12.0	7	3.6	0	0.0	0	0.0	0	0.0
Child less than 6 months	21	4.0	4	2.2	9	4.6	7	11.1	0	0.0	1	5.6
Child 6 months to 5 years	44	8.4	9	4.9	20	10.3	12	19.1	3	4.6	0	0.0
Pregnant	13	2.5	1	0.5	6	3.1	5	7.9	1	1.5	0	0.0
Home-based care	6	1.1	6	3.3	0	0.0	0	0.0	0	0.0	0	0.0
Other illness^**a**^	115	21.9	39	21.2	42	21.5	17	27.0	13	19.7	4	22.2
**Number of conditions per household**												
0^**b**^	90	17.1	17	9.2	51	26.2	4	6.4	15	22.7	3	16.7
1	263	50.0	82	44.6	101	51.8	37	58.7	34	51.5	9	50.0
2	132	25.1	64	34.8	34	17.4	14	22.2	15	22.7	5	27.8
3	41	7.8	21	11.4	9	4.6	8	12.7	2	3.0	1	5.6

CHW, community health worker.^a^Refers to any illness that is talked about but not listed as a specific condition or ‘other chronic’ in the tool, and include an illness that a patient has, fears having, is at risk of getting, or that the fieldworker is unclear about when the CHW and client are discussing it.^b^When the response to the routine health question “Are you sick?” was “no” and no other health need was identified.

### 3.2 Characteristics of the CHWs included in this study

The characteristics of the CHWs who were observed by trained fieldworkers during household visits are shown in Table 5. Overall, 34 CHWs were observed. The overall mean age (standard deviation [SD]) was 42.0 (9.9) years and ranged from 26 to 61 years. Facility C (50.5 [2.1] years) and D (50.5 [7.6] years) had the oldest CHWs, while facility B (35.3 [11.6] years) had the youngest. Only about a third (*n* = 11, 32%) of the 34 CHWs had completed secondary education (“matric” and above), and the overall mean [SD] length of CHW service was 7.65 (4.18) years (range, 1–17 years). Although most (*n* = 25, 73.6%) had attended the prescribed phase 1 WBPHCOT training, only 4 (5.9%) had passed, and of the four, only two had passed both phases 1 and 2.

**Table 5:** The characteristics of the CHWs.

**Characteristics**	**Overall**		**Facility A**		**Facility B**		**Facility C**		**Facility D**		**Facility E**	
**Number of CHWs observed n (%)**	34		12		10		2		4		6	
**Age (years)**												
Mean (SD)	42.00	9.93	45.75	6.55	35.30	11.59	50.50	2.12	50.50	7.55	37.17	6.43
Range	26–61		32–55		26–59		49–52		43–61		30–48	
**Education, *n* (%)**												
Some secondary	23	67.7	7	58.3	8	80.0	0	0.0	3	75.0	5	83.3
Completed secondary education (matric and above)	11	32.3	5	41.7	2	20.0	2	100.0	1	25.0	1	16.7
**Length of service as CHW (years)**												
Mean (SD)	7.65	4.18	11.33	3.85	6.90	2.38	7.00	0.00	1.00	0.00	6.17	0.75
Range	1–17		7–17		3–10		7–7		1–1		5–7	
**WBPHCOT training passed**												
None attended	9	26.5	1	8.3	0	0.0	0	0.0	3	75.0	5	83.3
Phase 1 attended not passed	21	61.8	11	91.7	10	100.0	0	0.0	0	0.0	0	0.0
Phase 1 attended & passed	2	5.9	0	0.0	0	0.0	1	50.0	1	25.0	0	0.0
Phase 1 & 2 passed	2	5.9	0	0.0	0	0.0	1	50.0	0	0.0	1	16.7

CHW, community health worker; SD, standard deviation; WBPHCOT, Ward-based Primary Healthcare Outreach Team.

### 3.3 Characteristics of the CHW visits (planned activities) to households

As shown in Table 6, duration of visit varied widely between CHWs but was similar across districts, with an overall mean of 13.5 (9.8; range 1–84) minutes. In all the facilities except facility C (19.1%), the CHWs mostly (65, 72, 65, and 78% for facilities A, B, D, and E, respectively) indicated ‘last week’ as their last visit to the households. For planned activities during the current visit, the most common plan, follow-up of chronic patients/medication delivery, was similar across facilities. On the other hand, the CHWs visited households much less to conduct household registration or check pregnant women.

**Table 6:** CHW activities.

**Characteristics**	**Overall**		**Facility A**		**Facility B**		**Facility C**		**Facility D**		**Facility E**	
**Number of CHW observed *n* (%)**	34		12		10		2		4		6	
**Number of household visited (*n*, %)**	376	100.0	147	39.1	124	32.8	40	10.6	51	13.6	14	3.7
**Visit duration (minutes)**												
Mean (SD)	13.54	9.80	11.22	8.62	11.49	9.34	20.10	8.86	19.00	10.55	17.36	9.04
Range	1–84		1–84		1–75		2–50		6–56		5–41	
**When CHW last visited this household, *n* (%)**												
Never	55	10.5	15	8.2	24	12.3	1	1.6	14	21.2	1	5.6
Last week	328	62.4	119	64.7	140	71.8	12	19.1	43	65.2	14	77.8
Last month	116	22.1	37	20.1	29	14.9	39	61.9	9	13.6	2	11.1
Within last 3 months to 1 year	27	5.1	13	7.1	2	1.0	11	17.5	0	0.0	1	5.6
**Planned activities for this visit, *n* (%)**												
Household registration	38	10.1	20	13.6	14	11.3	2	5.0	0	0.0	2	14.3
Follow-up of chronic patients/medication delivery	255	67.8	117	79.6	75	60.5	22	55.0	29	56.9	12	85.7
Check children/babies	34	9.0	10	6.8	12	9.7	12	30.0	0	0.0	0	0.0
Check pregnant women	10	2.7	2	1.4	1	0.8	7	17.5	0	0.0	0	0.0
Others^a^	71	18.9	12	8.2	23	18.6	8	20.0	27	52.9	1	7.1

CHW, community health worker; SD, standard deviation; WBPHCOT, Ward-based Primary Healthcare Outreach Team.^a^Other activities include defaulter tracing, non-sick older adult, referral follow-up, visiting the bedridden, providing health education, adherence support, and TB dotting.

### 3.4 Messages/activities scores of CHW visits by selected household and CHW characteristics

In Table 7, the overall mean messages/activities scores differed across facilities but were highest in facility E, at 51.6% (SD, 28.0%), and least in facility C, at 36.4% (SD, 24.2%). The SDs of the scores across all the facilities were wide (from 23.4% in facility A to 30.2% in facility B). CHWs who had completed secondary education (‘matric’ and above) had a higher overall mean messages/activities score (51.8% [SD, 27.5%]) compared to those who had received some secondary education (44.2% [SD, 26.5%]). CHWs messages/activities score was highest when CHWs had attended and passed phases 1 and 2 of the WBPHCOT training (*n* = 2, 100%) compared to when they had attended phase 1 and passed (48.3% [SD, 25.9%]) or attended phase 1 and failed (40.6% [SD, 21.3%]). The mean messages/activities scores were < 50.0% for all the planned activities (including household registration, follow-up of chronic patients/medication delivery, and checking children, babies, and pregnant women). The scores were also <50% when this was the first visit (last visit = never) or when the last visit occurred either 1 week ago (last week) or longer than 1 week ago. The scores were also <50% for all conditions except when visiting a pregnant person (53.3% [28.7%]).

**Table 7:** Messages/activities scores of CHW visits by selected household and CHW characteristics.

**Characteristics**	**Overall**		**Facility A**		**Facility B**		**Facility C**		**Facility D**		**Facility E**	
	**Mean score (%)**	**SD**	**Mean score (%)**	**SD**	**Mean score (%)**	**SD**	**Mean score (%)**	**SD**	**Mean score (%)**	**SD**	**Mean score (%)**	**SD**
**Overall score**	43.94	27.00	42.13	23.42	51.18	30.16	36.39	24.16	34.86	26.56	51.64	27.95
**CHW education**												
Some secondary education	44.2	26.50	50.02	29.27	37.92	18.03	–	–	17.78	16.78	66.25	26.89
Completed secondary education (matric and above)	51.85	29.21	45.83	8.33	20.00	0.00	65.00	49.50	33.33	0.00	100.00	0.00
**WBPHCOT training passed**												
None attended	43.54	31.55	30.00	0.00	–	–	–	–	17.78	16.78	66.25	26.89
Phase 1 attended not passed	40.59	21.30	49.72	21.59	30.55	16.62	–	–	–	–	–	–
Phase 1 attended and passed	48.33	25.92	–	–	–	–	66.67	0.00	30.00	0.00	–	–
Phase 1 and 2 passed	100.00	0.00	–	–	–	–	100.00	0.00	–	–	100.00	0.00
**When CHW last visited this household, *n* (%)**												
Never	37.93	33.35	19.26	18.61	72.48	27.64	25.00	0.00	43.33	39.70	–	–
Last week	46.40	25.62	44.94	22.73	51.76	27.78	36.31	26.33	36.86	25.03	53.96	26.19
Last month	37.91	27.11	50.14	21.47	33.63	37.21	32.07	24.37	18.48	16.18	25.00	0.00
Within last 3 months to 1 year	43.82	32.21	36.92	30.07	100.00	0.00	43.65	30.51	–	–	–	–
**Planned activities for this visit, *n* (%)**												
Household registration	42.46	25.62	37.71	15.69	55.71	36.90	32.11	29.93	–	–	28.57	0.00
Follow-up of chronic patients/medication delivery	44.22	25.25	45.35	23.51	48.59	27.12	32.86	21.34	34.57	23.52	53.29	28.24
Check children/babies	39.15	23.50	35.74	21.78	46.47	29.01	37.43	21.33	–	–	–	–
Check pregnant women	44.13	26.55	35.83	12.06	–	–	48.27	31.41	–	–	–	–
Others^a^	40.57	34.58	27.50	21.55	62.18	38.21	31.99	30.29	31.52	31.81	28.57	0.00
**Household conditions**												
Hypertension	41.77	22.98	42.41	20.44	47.69	27.41	25.59	18.89	34.37	22.47	45.10	25.23
Diabetes	29.92	15.10	27.97	10.22	35.67	15.65	22.24	12.31	20.52	8.42	45.21	30.23
HIV	44.18	28.53	44.64	22.30	49.45	34.74	39.20	26.37	39.56	25.40	–	–
Tuberculosis	23.26	26.99	34.00	38.76	20.89	23.43	–	–	14.58	17.18	–	–
Other chronic	33.39	25.53	26.78	26.02	50.93	22.90	25.74	10.45	26.28	21.32	100.00	0.00
Persistent cough	22.18	15.61	25.85	14.17	10.66	15.18	–	–	–	–	–	–
Child less than 6 months	49.11	20.36	46.43	27.04	51.09	18.67	51.02	21.60	–	–	28.57	0.00
Child 6 months to 5 years	33.86	18.21	38.15	15.71	37.92	16.83	28.61	20.58	15.00	13.23	–	–
Pregnant	53.30	28.74	50.00	0.00	62.50	34.46	48.57	25.20	25.00	0.00	–	–
Home-based care	41.85	9.73	41.85	9.73	–	–	–	–	–	–	–	–
Other illness	37.78	28.96	32.97	19.52	51.12	33.32	24.38	22.64	28.53	33.71	31.52	18.83
Routine check	43.95	38.89	45.70	23.82	50.05	31.83	36.39	24.16	51.64	27.95	34.18	26.74
**Number of conditions per household**												
1	53.95	28.97	54.32	26.16	60.10	30.62	45.06	25.21	41.76	29.51	64.29	27.98
2	30.25	12.78	32.44	12.00	32.03	12.53	24.47	15.21	19.73	8.68	38.00	7.58
3	20.70	8.82	24.09	7.12	19.12	11.88	17.18	5.94	13.64	6.43	6.06	0.00
**Household size**												
1	44.77	27.02	44.56	23.93	51.89	30.01	35.97	27.96	36.72	28.26	51.88	24.04
2	43.86	27.03	38.13	22.22	51.90	30.63	40.49	20.70	34.12	23.96	51.38	33.89
3	41.76	29.77	35.60	23.39	46.88	34.21	36.90	26.81	–	–	–	–
4	40.47	23.70	33.75	9.17	52.01	24.26	39.95	19.07	8.33	14.43	–	–
5	25.34	22.08	–	–	34.29	30.90	19.97	16.71	–	–	–	–

CHW, community health worker; SD, standard deviation; WBPHCOT, Ward-based Primary Healthcare Outreach Team.^a^Other activities include defaulter tracing, non-sick older adult, referral follow-up, visiting the bedridden, providing health education, adherence support, and TB dotting.

### 3.5 Multilevel model results of factors associated with the messages/activities scores

As shown in Table 8, the factors that are associated with the messages/activities score included CHW activity-related factors (visit duration and when the activity planned by CHW for that house on that day was checking on children and babies) and household-related factors (household size, number of conditions in a household, and the type of condition for which the CHW provided services).

**Table 8:** Results of fitting multilevel models for factors associated with the messages/activities scores.

**Variables**	**Estimated coefficients**	**SE**	**z**	**Confidence interval**		***p*-value**
				Lower	Upper	
***CHW activity*-*related factors***						
Visit duration (5-min change)	1.52	0.51	2.96	0.51	2.53	0.003**
Checking on children and babies	−10.11	3.12	−3.24	−16.22	−4.00	0.001**
Checking on pregnant women	11.43	6.17	1.85	−0.65	23.52	0.064
***Household-related factors***						
Household size (each extra person)	−3.63	1.05	−3.46	−5.69	−1.58	0.001**
Number of conditions in a household (each extra condition)	−21.65	1.77	−12.23	−25.12	−18.18	0.001**
**Conditions for which the CHW provided services**						
Hypertension	−5.14	2.50	−2.05	−10.04	0.23	0.040*
Diabetes	−15.52	2.88	−5.40	−21.16	−9.89	<0.001***
HIV	4.80	2.62	1.83	−0.33	9.93	0.067
**Tuberculosis (TB) and cough**						
TB alone no cough	−13.41	5.06	−2.65	−23.33	−3.49	0.008**
Cough alone no TB	−17.49	4.38	−4.38	−25.32	−9.66	<0.001***
Both TB and cough	9.39	18.25	0.51	−26.38	45.16	0.607

*p < 0.05; **p < 0.01; ***p < 0.001. We selected the model that minimized Akaike information criterion (AIC) as the best prediction model. SE, standard error; CHW, community health worker.

With each extra 5 min spent in the household and each additional message provided by the CHW, the messages/activities score increased by 1.5% (95% CI, 0.5 to 2.5, *p* = 0.003) and 9.2% (95% CI, 7.8 to 10.6, *p* < 0.001), respectively. For each extra person with HIV or when checking on pregnant women, the scores increased marginally by 4.8% (*p* = 0.067) and 11.4% (*p* = 0.064), respectively. However, for each extra person and condition in the household, the score decreased by 3.6% (95% CI, 1.6 to 5.7, *p* = 0.001) and 21.7% (95% CI, 18.2 to 25.1, *p* < 0.001), respectively. For each household where a member had hypertension, diabetes, TB alone, or cough alone, or when the CHWs planned to check on children and babies, the score also decreased by 5.1% (95% CI, 0.2 to 10.0, *p* < 0.040), 15.5% (95% CI, 9.9 to 21.2, *p* < 0.001), 13.4% (95% CI, 3.5 to 23.3, *p* = 0.008), 17.5% (95% CI, 9.7 to 25.3, *p* < 0.001), and 10.1% (95% CI, 4.0 to 16.2, *p* = 0.001), respectively. In multilevel modeling, no CHW-related characteristics were associated with the messages/activities score.

## 4 Discussion

We found evidence of very high frequencies of visits (twice a week and weekly) by CHWs to households, with >64.0% having occurred the previous week in most facilities. The planned activities were many, covering MCH, communicable and non-communicable diseases, although the most common was follow-up with chronic patients/delivery of medication. It is possible that the high frequency or regularity of CHW visits may be to prevent the loss of skills (51). In our previous study that reported on facilities A and B, frequent visits were because it was easier to go to households they knew. Furthermore, delivery of medications, one of their activities, required at least two monthly visits (to collect the household member’s card and deliver their medications) (48). Yet, after a “nurse mentor” (a qualified PN) had provided training and mentoring for CHWs and OTLs for over a year, fewer repeat visits were subsequently reported, and CHWs still managed to provide more comprehensive care (48).

Completing secondary education (‘matric’ or above) pre-employment and passing the phases of the WBPHCOT trainings seem to have some relevance with the messages/activities scores. Indeed, previous studies have reported better performance of CHW work for those with secondary education (42, 52). CHW background education is an important pre-selection criterion, and training, including relevant content to the CHW scope of work, is crucial if CHWs are to be effective in providing quality care (6, 53–56). In South Africa, several challenges have affected the successful implementation of the WBPHCOT training programs. These include the poor fit between training content and CHW skill gaps, lack or shortages of learning materials, inconvenient timing of training and non-supportive learning environment, and lack of budgeting (23). Thus, even if the CHWs had attended the training, most were yet to pass. Furthermore, newer CHWs with no related experience have been employed within the same programmatic constraints as previous CHWs (57). Furthermore, the framework to guide ongoing mentorship/supervision by CHW team leaders is reported to be underdeveloped (58). However, in this study, in the final multilevel model, the messages/activities scores no longer show an association with either education or CHW training.

In the multilevel models, messages/activities scores increased with each extra 5 min spent in the household. For each extra person, extra condition, and each household where a member had diabetes, TB alone, or cough alone, or when the CHWs planned to check on children and babies, the messages/activities score decreased. These findings may suggest a challenge in the balance between the number of conditions that a CHW may be required to address in a household and available time. While increasing the array of health topics covered by CHW is a tempting strategic approach to cover public health priorities, the present study shows that additional topics and less time are linked to decreased message scores. Beyond quality care, there is only so much a CHW can do when the number of conditions is high and time is short. Therefore, the quantity of time is key and may be an important consideration as an associated cost of CHW programs (59).

Other studies have also suggested better performance with longer visit duration. According to Goudge et al., in a South African district, during household registrations where a CHW was required to ask nine questions to elicit any health needs in a household, on average, CHWs were observed to have asked only four to five questions (43). Although the length of time spent was not reported in this previous study, it is assumed that the CHWs would have had to spend longer in the household to be able to ask all the relevant questions. In a qualitative study of household members, Diema Konlan et al. reported that 57% of participants (household members) indicated that the time spent in the household by the CHWs was either too short or that they wished that the CHWs had stayed longer, which corroborated the findings of an older study (60, 61). Further study is suggested in view of the scanty evidence regarding the influence of time spent and performance.

Evidence is lacking concerning the influence of household size on CHW performance, with existing evidence reporting the number of households covered by CHWs only (40). In our study, we found that increasing household size and increasing number of household conditions have a negative influence on messages/activities scores. It stands to reason to consider that it may be easy for a CHW to forget to give all the necessary messages to everyone with a condition in large households. To address this challenge within comprehensive care, individual CHW performance assessment on an ongoing basis is recommended to identify and strengthen individual-CHW-level issues (knowledge gap and challenges with adherence to stipulated guidelines) (30, 33).

In our study, the evidence suggested by the declining messages/activities scores for certain conditions, including diabetes, TB, cough, and children/babies, is important. In LMIC, CHWs are effective in providing promotive and preventive care for different conditions in MCH, as well as TB, hypertension, and diabetes (2). However, when CHWs must provide services for increasingly complex number of activities, it could result in poor performance (5). In South Africa, CHWs showed adequate confidence in giving prescribed medications and managing clients with diabetes but had low knowledge and confidence levels in managing clients with hypertension (38). Another previous study in six provinces of South Africa revealed that CHWs expressed the need for more training, especially on testing blood pressure and supporting diabetes, despite initial training (27). These findings emphasize the role of supportive supervision (62–64). Since increasing number of LMICs now prefer CHW programme that provide comprehensive care, program planners may consider utilizing available evidence when adding unto the wide array of tasks that CHWs perform.

### 4.1 Strength and limitation

We provided detailed quality of messages/activities scores and associated factors on several comprehensive care services provided by CHWs using a validated tool designed to investigate the process measures (compliance with standards of care) during household visits. The study also identified, with the use of the tool, variations in CHW competencies across settings and CHW teams. However, since the study did not examine community and health systems factors, interpretations of the findings are mostly limited to individual-level CHW characteristics and household factors. The influence of the additional burden due to the COVID-19 pandemic could not be considered in this study since the development and validation of the tool used in data collection pre-dated the pandemic. Finally, the small sample size in some facilities and conditions occasioned by the ongoing pandemic may have affected the robustness of the findings. Generalization of the results is limited to the CHW program for providing comprehensive care, particularly in LMICs.

## 5 Conclusion

In this study, additional time spent in the household increased the messages/activities score. Increasing household size; increasing number of household conditions; the presence of diabetes, TB alone, or cough alone; and CHWs having planned to check on children and babies are factors that led to decreased messages/activities scores. Our findings identified important characteristics that could form the focus of training and supportive supervision specific to these CHWs but replicable with adjustment of the tool to local contexts. The findings also support the relevance of ongoing assessments of CHW competency during household visits.

## Data availability statement

The original contributions presented in the study are included in the article/supplementary material, further inquiries can be directed to the corresponding author.

## Ethics statement

The studies involving humans were approved by The Human Research Ethics Committee (Medical) of the University of the Witwatersrand, Johannesburg (approval number M190933), the respective ethics committee of participating Ehlanzeni (MP-2020001-002) and Tshwane Districts Research Committees (GP-202001-012) approved this study protocol. The studies were conducted in accordance with the local legislation and institutional requirements. The participants provided their written informed consent to participate in this study.

## Author contributions

OB, JG, and FG conceptualized the study. JG and FG raised the funding. OB led the drafting of the manuscript and was responsible for overseeing data collection. OB, FG, and JL were responsible for the data analysis. OB, JG, FG, and JL contributed to drafting the manuscript. All authors contributed to the article and approved the submitted version.

## Footnotes

^1^ https://redcap.core.wits.ac.za/

## References

[1] Perry HB Chowdhury M Were M LeBan K Crigler L Lewin S. Community health workers at the dawn of a new era: 11. CHWs leading the way to “health for all”. Health Res Policy Syst. (2021) 19:1–21. doi: 10.1186/s12961-021-00755-5 34641891 PMC8506098

[2] Perry HB Zulliger R Rogers MM. Community health workers in low-, middle-, and high-income countries: an overview of their history, recent evolution, and current effectiveness. Annu Rev Public Health. (2014) 35:399–421. doi: 10.1146/annurev-publhealth-032013-182354, PMID: 24387091

[3] Perry H Zulliger R. How effective are community health workers? An overview of current evidence with recommendations for strengthening community health worker programs to accelerate progress in achieving the health-related millennium development goals. Baltimore: Johns Hopkins Bloomberg School of Public Health (2012).

[4] Scott K Beckham S Gross M Pariyo G Rao KD Cometto G. What do we know about community-based health worker programs? A systematic review of existing reviews on community health workers. Hum Resour Health. (2018) 16:1–17. doi: 10.1186/s12960-018-0304-x 30115074 PMC6097220

[5] Glenton C Javadi D Perry HB. Community health workers at the dawn of a new era: 5. Roles and tasks. Health Res Policy Syst. (2021) 19:1–16. doi: 10.1186/s12961-021-00748-4 34641903 PMC8506082

[6] Schleiff MJ Aitken I Alam MA Damtew ZA Perry HB. Community health workers at the dawn of a new era: 6. Recruitment, training, and continuing education. Health Res Policy Syst. (2021) 19:1–28. doi: 10.1186/s12961-021-00757-3 34641898 PMC8506097

[7] Freeman PA Schleiff M Sacks E Rassekh BM Gupta S Perry HB. Comprehensive review of the evidence regarding the effectiveness of community–based primary health care in improving maternal, neonatal and child health: 4. Child health findings. J Glob Health. (2017) 7:010904. doi: 10.7189/jogh.07.010904, PMID: 28685042 PMC5491948

[8] Mishra SR Neupane D Preen D Kallestrup P Perry HB. Mitigation of non-communicable diseases in developing countries with community health workers. Glob Health. (2015) 11 1:43–5. doi: 10.1186/s12992-015-0129-5, PMID: 26555199 PMC4641398

[9] Schneider H English R Tabana H Padayachee T Orgill M. Whole-system change: case study of factors facilitating early implementation of a primary health care reform in a south African province. BMC Health Serv Res. (2014) 14:1–11. doi: 10.1186/s12913-014-0609-y 25432243 PMC4261614

[10] Thomas LS Buch E Pillay Y. An analysis of the services provided by community health workers within an urban district in South Africa: a key contribution towards universal access to care. Hum Resour Health. (2021) 19:1–11. doi: 10.1186/s12960-021-00565-4 33602255 PMC7889710

[11] De Koning K Kok M Ormel H Kane S Rashid S Sarker M. A common analytical framework on factors influencing performance of close-to-community providers. KIT Royal Tropical Institute. (2014):78.

[12] Angwenyi V Aantjes C Kondowe K Mutchiyeni JZ Kajumi M Criel B. Moving to a strong(er) community health system: analysing the role of community health volunteers in the new national community health strategy in Malawi. BMJ Glob Health. (2018) 3:e000996. doi: 10.1136/bmjgh-2018-000996, PMID: 30498595 PMC6254745

[13] Kane S Kok M Ormel H Otiso L Sidat M Namakhoma I. Limits and opportunities to community health worker empowerment: a multi-country comparative study. Soc Sci Med. (2016) 164:27–34. doi: 10.1016/j.socscimed.2016.07.019, PMID: 27459022

[14] Taegtmeyer M Theobald S McCollum R Otiso L Mireku M de Koning K. Exploring perceptions of community health policy in Kenya and identifying implications for policy change. Health Policy Plan. (2015) 31:10–20. doi: 10.1093/heapol/czv007 25820367 PMC4724165

[15] Chin-Quee D Mugeni C Nkunda D Uwizeye MR Stockton LL Wesson J. Balancing workload, motivation and job satisfaction in Rwanda: assessing the effect of adding family planning service provision to community health worker duties. Reprod Health. (2016) 13:2. doi: 10.1186/s12978-015-0110-z 26732671 PMC4702334

[16] Bhutta Z Lassi Z Pariyo G Huicho L. Global experience of community health workers for delivery of health related millennium development goals: A systematic review, country case studies, and recommendation for integration into national health systems. Geneva: World Health Organization, Global Health Workforce Alliance (2010).

[17] Liu A Sullivan S Khan M Sachs S Singh P. Community health workers in global health: scale and scalability. Mount Sinai J Med. (2011) 78:419–35. doi: 10.1002/msj.20260 21598268

[18] Teklehaimanot HD Teklehaimanot A. Human resource development for a community-based health extension program: a case study from Ethiopia. Hum Resour Health. (2013) 11:1–12. doi: 10.1186/1478-4491-11-39, PMID: 23961920 PMC3751859

[19] Gopalan SS Mohanty S Das A. Assessing community health workers’ performance motivation: a mixed-methods approach on India's accredited social health activists (ASHA) programme. BMJ Open. (2012) 2:e001557. doi: 10.1136/bmjopen-2012-001557, PMID: 23019208 PMC3488714

[20] Soofi S Cousens S Turab A Wasan Y Mohammed S Ariff S. Effect of provision of home-based curative health services by public sector health-care providers on neonatal survival: a community-based cluster-randomised trial in rural Pakistan. Lancet Glob Health. (2017) 5:e796–806. doi: 10.1016/S2214-109X(17)30248-6, PMID: 28716351 PMC5762815

[21] Hafeez A Mohamud BK Shiekh MR Shah SAI Jooma R. Lady health workers programme in Pakistan: challenges, achievements and the way forward. J Pakistan Med Assoc. (2011) 61:210–5. PMID: 21465929

[22] National Department of Health of South Africa (2011). Provincial guidelines for the implementation of the three streams of PHC re-engineering Pretoria. Available at: http://www.jphcf.co.za/wp-content/uploads/2014/06/guidelines-for-the-implementation-of-the-three-streams-of-phc-4-Sept-2.pdf

[23] Jinabhai C Marcus T Chaponda A. Rapid appraisal of ward based outreach teams Pretoria: Albertina Sisulu Executive Leadership Programme in Health (2015). Available at: https://www.up.ac.za/media/shared/62/COPC/COPC%20Reports%20Publications/wbot-report-epub-lr-2.zp86437.pdf

[24] South African Department of Health. Policy framework and strategy for ward-based primary health care outreach teams 2018/19–2023/24. Pretoria, Republic of South Africa National Department of Health (2018). Available at: https://rhap.org.za/wp-content/uploads/2018/04/Policy-WBPHCOT-4-April-2018-1.pdf

[25] Vawda Y Gray A. South African health review 2018. Durban: Health Systems Trust (2018).

[26] Schneider H Sanders D Besada D Daviaud E Rohde S. Ward-based primary health care outreach teams in South Africa: developments, challenges and future directions. South African Health Rev. (2018) 2018:59–65.

[27] Murphy JP Moolla A Kgowedi S Mongwenyana C Mngadi S Ngcobo N. Community health worker models in South Africa: a qualitative study on policy implementation of the 2018/19 revised framework. Health Policy Plan. (2021) 36:384–96. doi: 10.1093/heapol/czaa172, PMID: 33367608 PMC8128020

[28] Babalola O Goudge J Levin J Brown C Griffiths F. Assessing the utility of a quality-of-care assessment tool used in assessing comprehensive care services provided by community health Workers in South Africa. Front Public Health. (2022) 10:10. doi: 10.3389/fpubh.2022.868252 PMC9149253 35651863

[29] Agarwal S Sripad P Johnson C Kirk K Bellows B Ana J. A conceptual framework for measuring community health workforce performance within primary health care systems. Hum Resour Health. (2019) 17:1–20. doi: 10.1186/s12960-019-0422-0 31747947 PMC6868857

[30] Kok M Crigler L Musoke D Ballard M Hodgins S Perry HB. Community health workers at the dawn of a new era: 10. Programme performance and its assessment. Health Res Policy Syst. (2021) 19:1–14. doi: 10.1186/s12961-021-00758-2 34641901 PMC8506096

[31] Hanefeld J Powell-Jackson T Balabanova D. Understanding and measuring quality of care: dealing with complexity. Bull World Health Organ. (2017) 95:368–74. doi: 10.2471/BLT.16.179309, PMID: 28479638 PMC5418826

[32] Donabedian A. The quality of care: how can it be assessed? JAMA. (1988) 260:1743–8. doi: 10.1001/jama.1988.03410120089033 3045356

[33] Kok MC Dieleman M Taegtmeyer M Broerse JE Kane SS Ormel H. Which intervention design factors influence performance of community health workers in low-and middle-income countries? A systematic review. Health Policy Plan. (2015) 30:1207–27. doi: 10.1093/heapol/czu126, PMID: 25500559 PMC4597042

[34] Yourkavitch J Zalisk K Prosnitz D Luhanga M Nsona H. How do we know? An assessment of integrated community case management data quality in four districts of Malawi. Health Policy Plan. (2016) 31:1162–71. doi: 10.1093/heapol/czw047, PMID: 27162235

[35] Agarwal A Gallo M Finlay A. Evaluation of the quality of community based integrated management of childhood illness and reproductive health programs in Madagascar: Prepared for the United States Agency for International Development through the President’s malaria initiative. Published for CDC by the USAID health care improvement project chevy chase. MD: University Research Co, LLC (2013).

[36] Gautham M Iyengar MS Johnson CW. Mobile phone–based clinical guidance for rural health providers in India. Health Informatics J. (2015) 21:253–66. doi: 10.1177/1460458214523153, PMID: 24621929

[37] Ashraf N Bandiera O Lee SS. Do-gooders and go-getters: career incentives, selection, and performance in public service delivery. Harvard Business School. (2015):1–60.

[38] Moetlo GJ Pengpid S Peltzer K. An evaluation of the implementation of integrated community home-based care services in Vhembe District, South Africa. Indian J Palliat Care. (2011) 17:137. doi: 10.4103/0973-1075.84535 21976854 PMC3183603

[39] Laurenzi CA Gordon S Skeen S Coetzee BJ Bishop J Chademana E. The home visit communication skills inventory: piloting a tool to measure community health worker fidelity to training in rural South Africa. Res Nurs Health. (2020) 43:122–33. doi: 10.1002/nur.22000, PMID: 31793678

[40] Kawakatsu Y Sugishita T Tsutsui J Oruenjo K Wakhule S Kibosia K. Individual and contextual factors associated with community health workers’ performance in Nyanza Province, Kenya: a multilevel analysis. BMC Health Serv Res. (2015) 15:1–10. doi: 10.1186/s12913-015-1117-4 26429072 PMC4589910

[41] Kelkar S Mahapatro M. Community health worker: a tool for community empowerment. Health Popul Perspect Issues. (2014) 37:57–65.

[42] Njororai F Ganu D Nyaranga KC Wilberforce C. Role of socio-demographic and environmental determinants on performance of community health Workers in Western Kenya. Int J Environ Res Public Health. (2021) 18:11707. doi: 10.3390/ijerph182111707, PMID: 34770222 PMC8582826

[43] Goudge J de Kadt J Babalola O Muteba M Tseng Y-h Malatji H. Household coverage, quality and costs of care provided by community health worker teams and the determining factors: findings from a mixed methods study in South Africa. BMJ Open. (2020) 10:e035578. doi: 10.1136/bmjopen-2019-035578, PMID: 32819939 PMC7440700

[44] Griffiths F Babalola O Brown C de Kadt J Malatji H Thorogood M. Development of a tool for assessing quality of comprehensive care provided by community health workers in a community-based care programme in South Africa. BMJ Open. (2019) 9:e030677. doi: 10.1136/bmjopen-2019-030677, PMID: 31492789 PMC6731907

[45] Sedibeng District Profile and Analysis. District development model (2020). Available at: https://www.cogta.gov.za/ddm/wp-content/uploads/2020/11/Sedibeng-October-2020.pdf

[46] Tshwane District Profile and Analysis. District development model (2020). Available at: https://www.cogta.gov.za/ddm/wp-content/uploads/2020/08/2nd-Take_Final_DistrictProfile_TSHWANE2306-1-002

[47] Ehlanzeni District Profile and Analysis. District development model (2020). Available at: https://www.cogta.gov.za/ddm/wp-content/uploads/2020/07/Take3_Final-Edited-Ehlanzeni-DM_07July2020-FINAL.pdf (Accessed December 22, 2022)

[48] Goudge J Babalola O Malatji H Levin J Thorogood M Griffiths F. The effect of a roving nurse mentor on household coverage and quality of care provided by community health worker teams in South Africa: a longitudinal study with a before, after and 6 months post design. BMC Health Serv Res. (2023) 23:1–11. doi: 10.1186/s12913-023-09093-4 36814259 PMC9948528

[49] National Department of Health. National Department of health annual report 2017/2018 Pretoria. South Africa: South Africa National Department of Health (2019).

[50] Royston P Ambler G Sauerbrei W. The use of fractional polynomials to model continuous risk variables in epidemiology. Int J Epidemiol. (1999) 28:964–74. doi: 10.1093/ije/28.5.964 10597998

[51] Chaudhary N Mohanty P Sharma M. Integrated management of childhood illness (IMCI) follow-up of basic health workers. Indian J Pediatrics. (2005) 72:735–9. doi: 10.1007/BF02734143, PMID: 16186673

[52] Crispin N Wamae A Ndirangu M Wamalwa D Wangalwa G Watako P. Effects of selected socio-demographic characteristics of community health workers on performance of home visits during pregnancy: a cross-sectional study in Busia District, Kenya. Global J Health Sci. (2012) 4:78. doi: 10.5539/gjhs.v4n5p78 PMC4776911 22980380

[53] World Health Organization. Handbook for national quality policy and strategy: A practical approach for developing policy and strategy to improve quality of care. Geneva: World Health Organization; Licence (2018).

[54] Perry H Crigler L Hodgins S. Developing and strengthening community health worker programs at scale: A reference guide for program managers and policy makers. Dhaka, Bangladesh: University Press Ltd. (2013).

[55] World Health Organization. WHO guideline on health policy and system support to optimize community health worker programmes. Geneva: World Health Organization (2018). 30431747

[56] World Health Organization. Delivering quality health services: A global imperative for universal health coverage. Geneva: World Health Organization, Organisation for Economic Co-operation and Development, and The World Bank (2018).

[57] Nyalunga S Ndimande J Ogunbanjo G Masango-Makgobela A Bogongo T. Perceptions of community health workers on their training, teamwork and practice: a cross-sectional study in Tshwane district, Gauteng. South Africa South African Family Pract. (2019) 61:144–9. doi: 10.1080/20786190.2019.1613061

[58] Assegaai T Schneider H. National guidance and district-level practices in the supervision of community health workers in South Africa: a qualitative study. Hum Resour Health. (2019) 17:1–10. doi: 10.1186/s12960-019-0360-x 30943986 PMC6446406

[59] Masis L Gichaga A Zerayacob T Lu C Perry HB. Community health workers at the dawn of a new era: 4. Programme financing. Health Res Policy Syst. (2021) 19. doi: 10.1186/s12961-021-00751-9 PMC8506106 34641893

[60] Diema Konlan K Kossi Vivor N Gegefe I Imoro AA-R Esinam Kornyo B Peter Kwao I. The practice of home visiting by community health nurses as a primary healthcare intervention in a low-income rural setting: a descriptive cross-sectional study in the Adaklu District of the Volta region, Ghana. Sci World J. (2021) 2021:1–11. doi: 10.1155/2021/8888845, PMID: 33833622 PMC8012147

[61] Amonoo-Lartson R De Vries J. Patient care evaluation in a primary health care programme: the use of tracer conditions as a simple and appropriate technology in health care delivery∗. Soc Sci Med. (1981) 15:735–41. doi: 10.1016/0271-7123(81)90096-1 6976632

[62] Kok MC Vallières F Tulloch O Kumar MB Kea AZ Karuga R. Does supportive supervision enhance community health worker motivation? A mixed-methods study in four African countries. Health Policy Plan. (2018) 33:988–98. doi: 10.1093/heapol/czy082, PMID: 30247571 PMC6263021

[63] Madede T Sidat M McAuliffe E Patricio SR Uduma O Galligan M. The impact of a supportive supervision intervention on health workers in Niassa, Mozambique: a cluster-controlled trial. Hum Resour Health. (2017) 15:1–11. doi: 10.1186/s12960-017-0213-4 28865466 PMC5581457

[64] Singh D Negin J Orach CG Cumming R. Supportive supervision for volunteers to deliver reproductive health education: a cluster randomized trial. Reprod Health. (2016) 13:1–10. doi: 10.1186/s12978-016-0244-7 27716313 PMC5048471

[65] Cometto G Ford N Pfaffman-Zambruni J Akl EA Lehmann U McPake B. Health policy and system support to optimise community health worker programmes: an abridged WHO guideline. Lancet Glob Health. (2018) 6:e1397–404. doi: 10.1016/S2214-109X(18)30482-0, PMID: 30430994

[66] Degefie T Marsh D Gebremariam A Tefera W Osborn G Waltensperger K. Community case management improves use of treatment for childhood diarrhea, malaria and pneumonia in a remote district of Ethiopia. Ethiop J Health Dev. (2009) 23. doi: 10.4314/ejhd.v23i2.53227

[67] Kalyango JN Rutebemberwa E Alfven T Ssali S Peterson S Karamagi C. Performance of community health workers under integrated community case management of childhood illnesses in eastern Uganda. Malar J. (2012) 11:1–13. doi: 10.1186/1475-2875-11-282 22905758 PMC3480882

[68] Kelly JM Osamba B Garg RM Hamel MJ Lewis JJ Rowe SY. Community health worker performance in the management of multiple childhood illnesses: Siaya District, Kenya, 1997–2001. Am J Public Health. (2001) 91:1617–24. doi: 10.2105/AJPH.91.10.1617, PMID: 11574324 PMC1446843

[69] Mukanga D Babirye R Peterson S Pariyo G Ojiambo G Tibenderana J. Can lay community health workers be trained to use diagnostics to distinguish and treat malaria and pneumonia in children? Lessons from rural Uganda. Tropical Med Int Health. (2011) 16:1234–42. doi: 10.1111/j.1365-3156.2011.02831.x, PMID: 21752163

[70] Yeboah-Antwi K Pilingana P Macleod WB Semrau K Siazeele K Kalesha P. Community case management of fever due to malaria and pneumonia in children under five in Zambia: a cluster randomized controlled trial. PLoS Med. (2010) 7:e1000340. doi: 10.1371/journal.pmed.1000340, PMID: 20877714 PMC2943441

